# Development and Application of Gene-Specific Markers for Tomato Yellow Leaf Curl Virus Resistance in Both Field and Artificial Infections

**DOI:** 10.3390/plants10010009

**Published:** 2020-12-23

**Authors:** Jang Hee Lee, Dae Jun Chung, Je Min Lee, Inhwa Yeam

**Affiliations:** 1 Department of Horticultural Science, Kyungpook National University, Daegu 41566, Korea; wkzz8282@gmail.com (J.H.L.); quatez11@gmail.com (D.J.C.); 2 Department of Horticulture and Breeding, Andong National University, Andong 36729, Korea

**Keywords:** tomato, TYLCV, resistance gene, marker-assisted selection, gene-pyramiding

## Abstract

Tomato yellow leaf curl virus (TYLCV) is a disease that is damaging to tomato production worldwide. Resistance to TYLCV has been intensively investigated, and single resistance genes such as *Ty-1* have been widely deployed in breeding programs. However, resistance-breaking incidences are frequently reported, and achieving durable resistance against TYLCV in the field is important. In this study, gene-specific markers for *Ty-2* and *ty-5*, and closely-linked markers for *Ty-4* were developed and applied to distinguish TYLCV resistance in various tomato genotypes. Quantitative infectivity assays using both natural infection in the field and artificial inoculation utilizing infectious TYLCV clones in a growth chamber were optimized and performed to investigate the individual and cumulative levels of resistance. We confirmed that *Ty-2* could also be an effective source of resistance for TYLCV control, together with *Ty-1*. Improvement of resistance as a result of gene-pyramiding was speculated, and breeding lines including both *Ty-1* and *Ty-2* showed the strongest resistance in both field and artificial infections.

## 1. Introduction

Tomato (*Solanum lycopersicum*) is a model fruit-bearing plant and is the horticultural crop with the highest economic importance worldwide [1]. Many viral diseases affect the development and vegetative growth of tomatoes [2]. Tomato yellow leaf curl virus (TYLCV) is one of the viruses that cause the highest economic damage to the tomato industry [3]. TYLCV symptoms present initially in young leaves, and symptoms progress as the leaves age. Symptoms include yellowing, curling, and cupping of leaves, ultimately leading to decreased photosynthetic efficiency [4]. The resultant stunting and abortion of flowering can significantly decrease fruit yield [4].

Under natural circumstances, TYLCV infection is transmitted by different whiteflies within the *Bemisia tabaci* complex [5]. The first report of tomato damage attributed to TYLCV came from Israel in 1959 [6]. *B. tabaci* complex transmits members of five plant virus groups including Begomovirus [7,8]. Most of the harmful whiteflies worldwide are of *B. tabaci *Middle East Asian Minor 1 (MEAM1) and *B. tabaci* MEDITERRANEAN (MED) [8]. The management of TYLCV relies heavily on insecticide treatments for the control of whiteflies. However, due to the invasiveness and the frequent circulation of the virus, such chemical controls often fail, allowing further spread of the disease [9]. Heavy application of pesticides may also produce environmental pollution [10]. Physical methods such as fine-mesh screens are used in some regions. However, physical barriers increase the cost of production and act only to prevent the access of whiteflies to tomato plants without reducing the size of whitefly populations [11,12]. It is very difficult to exterminate whiteflies because they have an extremely wide host range [13]. To obtain reliable and durable resistance against TYLCV, tomato breeding programs have utilized genetic sources that carry TYLCV resistance or tolerance. The introduction of genetic resistance in tomato breeding programs has been effective in preventing losses in yield due to TYLCV, and it can reduce the cost of controlling disease [14].

The tomato genome contains multiple TYLCV resistance loci. The first resistance gene reported was *Ty-1*, which comes from *S. chilense* LA1969 and is located on chromosome 6 [15]. Another resistance gene, *Ty-3*, which originated in *S. chilense* LA1932, is also located on chromosome 6 [16]. *Ty-1* and *Ty-3* are allelic and encode an RNA-dependent RNA polymerase [17]. *Ty-1/3* is known to increase the cytosine methylation of viral genomes, and plants can acquire TYLCV resistance [18]. The gene *Ty-2* originated in *S. habrochaites* “B6013”, located on the long arm of chromosome 11 [19]. Recently, *Ty-2* has been identified as the nucleotide-binding domain and leucine-rich repeat-containing (NB-LRR) gene [20]. The gene *Ty-4* from *S. chilense* LA1932 maps to chromosome 3 and has been reported to increase resistance levels in combination with *Ty-3* [21,22]. The recessive TYLCV resistance gene *ty-5* from *S. peruvianum* resides on chromosome 4 and encodes the mRNA surveillance factor *Pelota* [23]. *Ty-6*, which has been mapped to chromosome 10, is effective in complementing the resistance conferred by *Ty-3* and *ty-5* [24]. Additionally, *Ty-6* confers resistance to tomato mottle virus (ToMoV), suggesting that *Ty-6* controls both mono- and bi-partite begomoviruses in tomatoes [24].

Tomato breeding strategies have focused primarily on introgressing genes of interest from related wild germplasm. The introduction of genetic resistance against several diseases has proven successful in commercial tomato breeding programs [25]. Although multiple TYLCV resistance genes have been investigated, most commercially available tomato cultivars have a single TYLCV resistance gene, usually *Ty-1/3*. The occurrence of *Ty-1/3* resistance-breaking TYLCV strains, or outbreaks of *Ty-1/3* resistance due to specific environmental conditions, has repeatedly been reported [26,27]. Therefore, successive introgression of multiple resistance genes is necessary to accomplish durable and reliable resistance against TYLCV. The use of marker-assisted selection (MAS) to track each target gene is essential for effective resistance-gene-pyramiding programs.

We developed gene-specific markers for *Ty-2* and *ty-5*, and also closely-linked markers targeting *Ty-4* resistance. These newly developed markers for *Ty-2*, *Ty-4*, and *ty-5*, in addition to the *Ty-1/3* gene-specific marker reported previously by Jung et al. [28] were applied to distinguish TYLCV resistance in various tomato genotypes, including commercial cultivars. To explore the resistance level of each gene and analyze the effect of resistance-gene-stacking, quantitative infectivity assays using both natural infection in the field and artificial inoculation utilizing infectious TYLCV clones in a growth chamber were optimized and applied. A positive effect from combining multiple TYLCV resistances was observed in several cases, emphasizing the necessity for MAS and resistance-gene-pyramiding in TYLCV resistance breeding programs.

## 2. Results

### 2.1. Development of Gene-Specific Markers for Ty-2, Ty-4, and ty-5 Resistances

**Figure 1:**
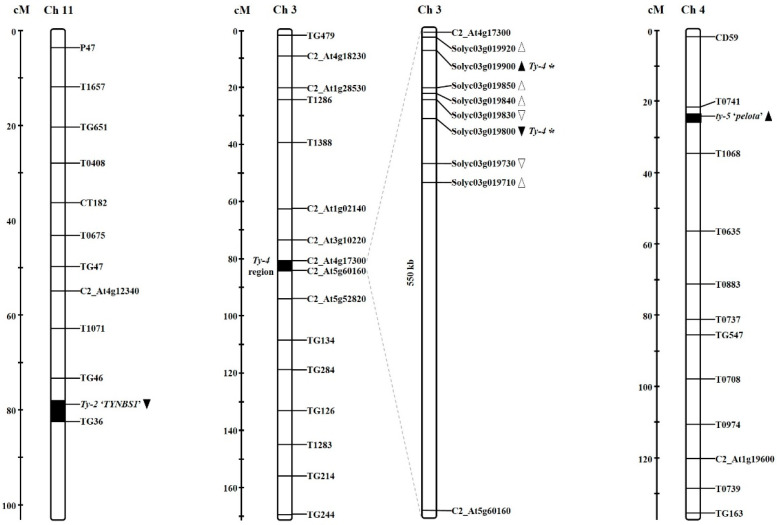
Genetic map of the begomovirus resistance locus on tomato chromosomes. Bars with the molecular markers indicate data simplified from the Tomato-EXPEN 2000 map (Sol Genomics Network, SGN, 2019). Closed arrowheads indicate the location of resistance genes that were used in marker development. Open arrowheads indicate possible *Ty-4* candidate genes. * *Ty-4* closely-linked marker.

Of the six TYLCV resistance loci identified (*Ty-1* to *Ty-6*), molecular markers for *Ty-2*, *Ty-4*, and *ty-5* resistance were developed and utilized in this study. The *Ty-2* and *ty-5* resistance genes, located on chromosome 11 and 4, respectively, were characterized [20,23], allowing the development of sequence-based markers. In the case of *Ty-4*, we focused on candidate genes located within a 550 kb interval on chromosome 3 [21] and generated closely-linked markers (Figure 1). All the markers developed in this study were tested plant materials including known resistance sources for *Ty-2*, *Ty-4*, and *ty-5* resistances (Supplementary Figure S2)

**Figure 2:**
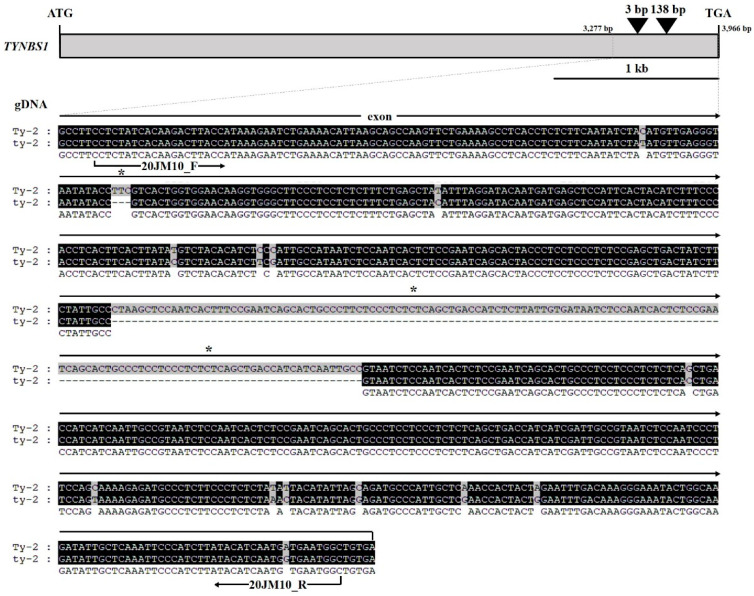
Representation of insertion or deletion (indel) information for *Ty-2* (TYNBS1) gene-based marker development. Schematic representation of the *Ty-2* (TYNBS1) gene structure with exons and intron. The black boxes indicate multiple sequence alignments of a portion of the cloning results between the susceptible and resistant varieties. Position and sequence information for forward and reverse primers used for marker development is indicated. Asterisks indicate positions of the 3 bp and 138 bp insertions used for closely-linked marker sites used for indel markers.

The *Ty-2* gene, also known as TYNBS1, encodes a nucleotide-binding domain and a leucine-rich repeat-containing (NB-LRR) protein [20]. TYNBS1 is located upstream of Solyc11g069660 [20]. Comparative sequence analysis of susceptible and resistant alleles at the *Ty-2* locus identified 47 single nucleotide polymorphisms (SNPs) and two insertions (data not shown). An insertion or deletion (indel) marker was developed based on two insertions: a 3 bp insertion at position 2109 and a 138 bp insertion at position 2309 of the genic region (Figure 2).

**Figure 3:**
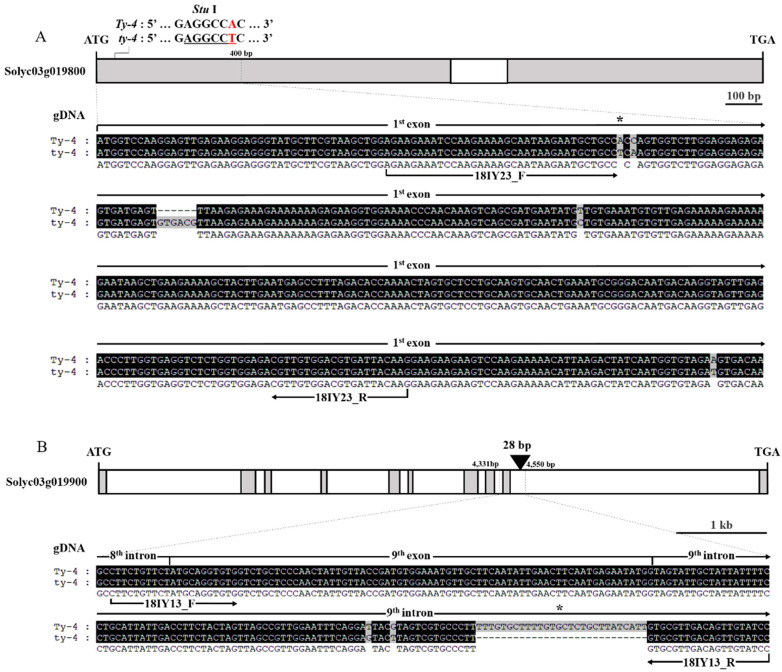
Representation of single nucleotide polymorphism (SNP) and indel information for *Ty-4* closely-linked markers. (**A**) Schematic representation of the Solyc03g019800 gene structure with exons and intron. (**B**) Schematic representation of the Solyc03g019900 gene structure with exons and introns. The black boxes indicate multiple sequence alignments of the portion of the cloning results between the susceptible and resistant varieties. White boxes indicate coding sequences (exons) and solid lines indicate non-coding sequences (introns). Asterisks indicate positions of SNP and 28 bp insertions used for closely-linked marker sites used for dCAPS (*Stu* I) and indel markers.

The resistance gene *Ty-4* exists within the 550 kb region between C2_At4g17300 and C2_At5g60160 on chromosome 3 [21]. To generate an efficient marker for *Ty-4* resistance, we focused on eight genes associated with disease resistance in this region: Solyc11g019710, Solyc11g019730, Solyc11g019800, Solyc11g019830, Solyc11g019840, Solyc11g019850, and Solyc11g019850. We identified markers closely-linked to Solyc11g019800 and Solyc11g019900. Sequence analysis of susceptible and resistant alleles at the Solyc11g019800 locus identified 10 SNPs (Supplementary Figure S1). A derived-cleaved amplified polymorphic sequence (dCAPS) marker was developed based on an SNP (T/A) located at position 79 of exon 1 (Figure 3A). The SNP polymorphism caused a single amino acid change from serine to threonine. Comparative sequence analysis of susceptible and resistant alleles at the Solyc11g019900 locus identified 13 SNPs, two insertions, and one deletion (data not shown). An indel marker was developed based on a 28 bp insertion located at position 4503 in intron 9 (Figure 3B).

**Figure 4:**
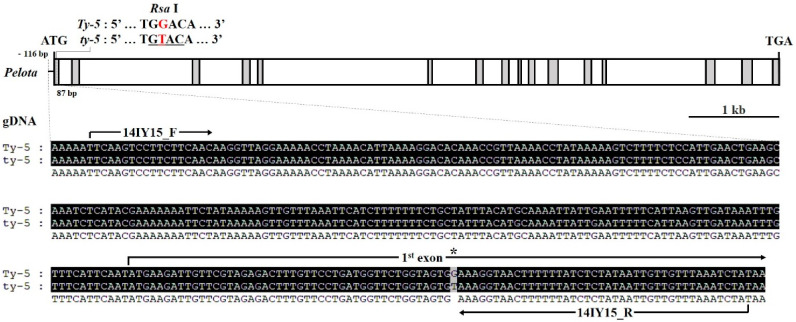
SNP information for *ty-5* (*Pelota*) gene-based marker development. Schematic representation of the *ty-5* (*Pelota*) gene structure with exons and introns. The black boxes indicate multiple sequence alignments of the portion of cloning results between the susceptible and resistant varieties. White boxes indicate coding sequences (exons) and solid lines indicate non-coding sequences (introns). Asterisks indicate positions of SNPs used for gene-based markers and restriction endonuclease sites (*Rsa* I) used for derived-cleaved amplified polymorphic sequence (dCAPS) markers.

A recessive resistant gene, *ty-5*, in the breeding line TY172, derived by introgression from *S. peruvianum*, encodes a messenger RNA surveillance factor, pelota, involved in ribosome recycling [23]. We retrieved the sequence of *ty-5* from the TY172 resistance line and compared it with the sequence of the susceptible reference. The gene is 8178 bp long and is composed of 16 exons and 15 introns with a single SNP (NCBI GenBank Accession No. KC447287.1 and Figure 4). A dCAPS marker was developed based on an SNP (T/G) located at the position 47 in exon 1 (Figure 4). The SNP polymorphism results in a single amino acid change of glycine to valine [29].

### 2.2. Application of Molecular Markers

**Figure 5:**
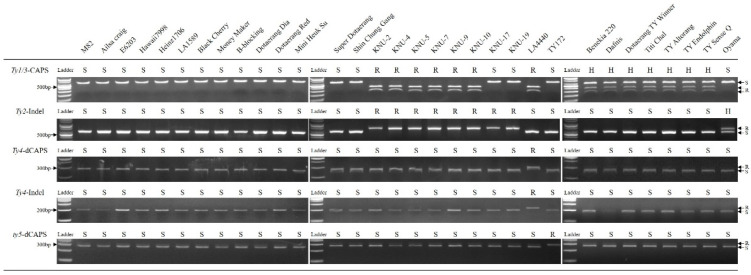
Analysis of gene-specific markers for tomato yellow leaf curl virus (TYLCV) resistances in inbred lines and commercial cultivars. R indicates resistant genotype, S indicates susceptible genotype, and H indicates heterozygous genotype. A *Ty-1/3* marker is designed as a CAPS marker, a *Ty-2* marker is designed as indel markers, a *ty-5* marker is designed as a dCAPS marker, and indel and dCAPS markers are designed for *Ty-4* resistance.

To determine the TYLCV resistance of each plant genotype, genetic markers designed for *Ty-1/3*, *Ty-2*, *Ty-4*, and *ty-5* resistance genes were applied to the 32 cultivars used in this study. Ten out of the 17 accessions and 8 out of 15 commercial varieties carried more than one resistance gene against TYLCV (Figure 5). None of the accessions or commercial cultivars expected to be susceptible carried any known TYLCV resistance gene. Most of the breeding lines developed for TYLCV resistance, except for KNU-17 and 19, carried both the *Ty-1/3* and *Ty-2* resistance genes. Most commercial cultivars that had been claimed by seed companies to be TYLCV resistant were heterozygous for *Ty-1/3* resistance, except the ‘Oyama’ cultivar, which had *Ty-2* resistance. The two *Ty-4* markers designed based on the two genes located within the 550 kb region of *Ty-4* resistance were effective in identifying LA4440, which is known to contain *Ty-4* resistance in addition to *Ty-3* resistance [21]. The selection efficiency of these two markers was not determined in this study because the markers were not tested on segregating population for *Ty-4* resistance.

### 2.3. Disease Evaluation Using Natural Infection

**Figure 6:**
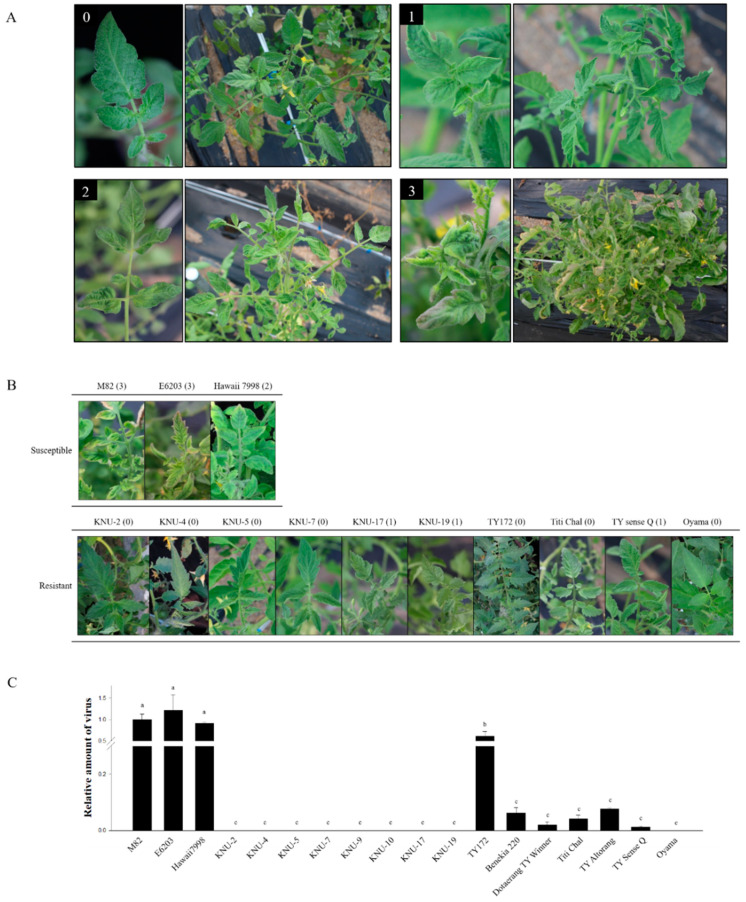
Infectivity assay results from natural infection in the field. (**A**) TYLCV disease severity scales for tomatoes naturally infected in the field. The number on each photograph indicates the symptom score. 0 = no visible symptom, resistant; 1 = light leaf yellowing of the leaflet margins, resistant; 2 = moderate plant stunting with leaf yellowing and curling, susceptible; 3 = severe plant stunting with leaf curling and yellowing and cessation of plant growth, susceptible. (**B**) Phenotypic evaluation six weeks post-transplantation. Three plants per genotype were used for randomly distributed replicates. TYLCV disease severity is indicated in brackets for each sample. (**C**) TYLCV bioassay detecting relative amount of TYLCV gene fragments in natural infection. DNA extracted from a young leaf six weeks after transplantation was used for quantitative real-time PCR (qPCR). All data from qPCR were merged after normalization. a, b and c indicate significant difference at *p* ≤ 0.05, respectively, by Duncan’s multiple range test.

Natural infection by whitefly was carried out in the field at Kyungpook National University (Daegu, Korea). Phenotypes were observed and evaluated for six weeks after transplanting. Disease severity following natural infection was assessed using a scale from 0 to 3 (Figure 6A,B). Based on phenotypic evaluation using natural infection, resistance genotypes with any known resistance genes were clearly distinguishable from the susceptible genotypes. To measure the amount of virus in the plants, quantitative real-time PCR was used to detect the relative amounts of virus (Figure 6C). Leaf samples were collected from the apical parts of young leaves six weeks after transplanting. A set of primers was designed to amplify viral gene fragment to detect viral accumulation (Supplementary Table S1). Three susceptible genotypes, M82, E6203, and Hawaii7998, showed high levels of viral DNA accumulation. Viral gene amplification was not detected or was slightly detected at non-significant levels in all resistance genotypes containing either one or both of *Ty-1* and *Ty-2*. TY172, containing the recessive resistance gene *ty-5* with no symptom development in phenotypic evaluation, showed amplification of viral gene, although it is significantly lower than all other susceptible genotypes and significantly higher than the rest of the resistance genotypes. The relative viral amounts, as evaluated by qPCR, were found to be consistent with the visual phenotyping results in most cases. These results showed that relative viral amounts were clearly distinguishable between the susceptible and resistant genotypes.

### 2.4. Disease Evaluation Using Infectious Clones

**Figure 7:**
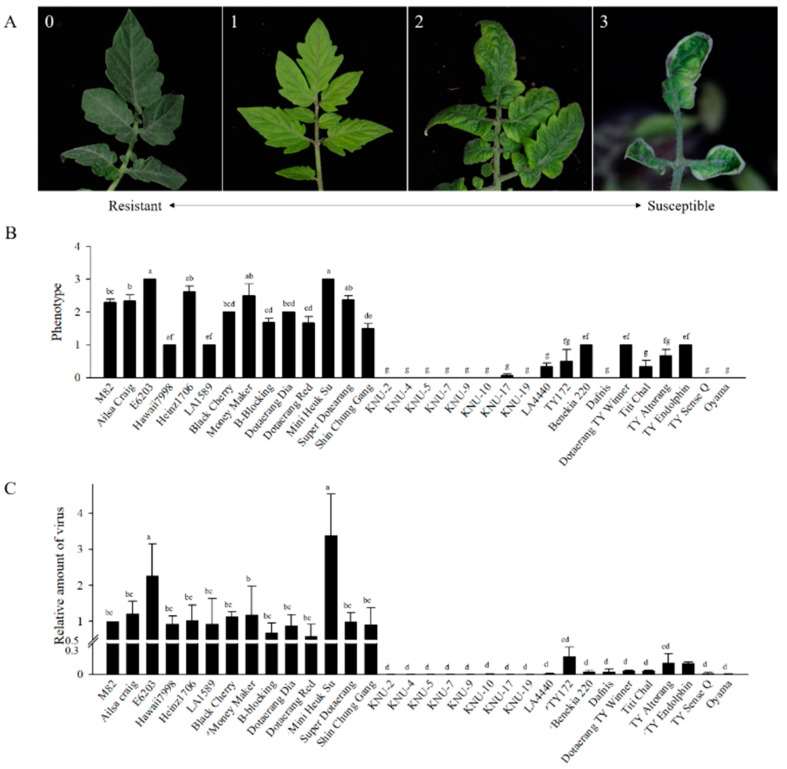
Infectivity assay of the TYLCV infectious clone via Agrobacterium–mediated inoculaTable 82. was used as the susceptible control. (**A**) TYLCV disease severity using agro-mediated inoculation of various tomato genotypes. The number on each photograph indicates symptom score. 0 = no visible symptom, resistant; 1 = very slight yellowing of leaflet margins of apical leaf, resistant; 2 = some yellowing and minor curling of leaflet ends, susceptible; 3 = wide range of leaf yellowing, curling, and cupping, susceptible. Score 4 was not observed under growth chamber conditions. (**B**) Phenotypic rating five weeks after inoculation. (**C**) Relative amount of virus in plants after agro-mediated infection. DNA extracted from young leaves five weeks post-inoculation was used for quantitative real-time PCR (qPCR). All data from qPCR were merged after normalization. a, b, c, d, e, f and g indicate significant difference at *p* ≤ 0.05, respectively, by Duncan’s multiple range test.

Agro-mediated inoculation was used to perform inoculation with the infectious TYLCV clone, and plants were evaluated five weeks after inoculation. The stunted growth observed in naturally infected tomatoes in the field was not detected in artificially infected tomatoes. Considering differing inoculation procedures and growing conditions between natural and artificial infections, a different disease measuring scale was applied to each set of plants. Disease severity was determined following a 0 to 4 disease severity index (DSI) scale, as described previously by Friedmann et al. [30] (Figure 7A). Visible differences in phenotypic outcomes were detected between susceptible and resistant groups. Fourteen susceptible genotypes were assessed and most plants had a disease scale of 2 or 3, with the exception of the two accessions: Hawaii7998 (*S. lycopersicum*) and LA1589 (*S. pimpinellifolium*) (Figure 7B). Both Hawaii7998 and LA1589 had a scale of 1 (resistant). No visible symptoms (scale 0) or very slight chlorosis (scale 1) was observed in 18 resistant genotypes. No development of symptoms was detected in any of the eight breeding lines (KNU lines). LA4440 (*Ty-1/3* and *Ty-4*) and TY172 (*ty-5*) showed relatively weak symptoms, although there were no statistically significant differences. Seven commercial varieties heterozygous for *Ty-1/3* resistance were evaluated. Although most of the seven commercial varieties retained resistance relative to the susceptible genotypes, some of the commercial varieties showed weak symptom development. A commercial cultivar, ‘Oyama’, which is heterozygous for *Ty-2* resistance, showed no visible symptoms.

The relative viral amounts, as evaluated by qPCR, were found to be consistent with the visual phenotyping results (Figure 7C). These results showed that relative viral amounts were clearly distinguishable between the susceptible and resistant genotypes.

## 3. Discussion

### 3.1. Genotyping of TYLCV Resistances with Molecular Markers

Thirty-two genotypes were evaluated using genetic markers for *Ty-1/3*, *Ty-2*, *Ty-4*, and *ty-5* resistance, and gene-specific markers for *Ty-1/3*, *Ty-2*, and *ty-5* resistance, in addition to a closely-linked marker for *Ty-4* resistance. These markers included the four markers developed in this study and a previously developed marker for *Ty-1/3*. *Ty-1* and *Ty-3* have been confirmed to be allelic [17], and the marker used for *Ty-1/3* in this study can distinguish *Ty-1* and *Ty-3* resistance from susceptibility. Eight commercial cultivars claimed to be resistant to TYLCV were included, and we confirmed that most of them were heterozygous for *Ty-1/3* resistance. The exception was ‘Oyama’, which was heterozygous for *Ty-2* resistance. *Ty-1* was the first resistance gene identified in tomato and confers resistance to TYLCV [15]. *Ty-2* was discovered approximately 15 years later, a delay and rather narrower range resistance, which explains its infrequent use in commercial breeding programs [31,32].

The markers developed for *Ty-4* were designed based on the DNA polymorphisms in Solyc11g019800 and Solyc11g019900, which are located in the 550 kb interval, which also includes *Ty-4* resistance on chromosome 3 [21]. The newly developed *Ty-4* markers were able to distinguish genotypes with *Ty-4* resistance and ascertained that *Ty-4* was not introgressed into any other breeding lines or commercial cultivars tested in this study. Because the markers were not tested on a segregating population for *Ty-4* resistance in this study, it would be interesting to determine the selection accuracy of these markers, which could be enabled by the development of segregating population and precise phenotyping of *Ty-4* resistance. Gene-specific markers for *ty-5* were able to differentiate TY172, which is derived from *S. peruvianum*, for *ty-5* resistance [33]. No other genotypes contained *ty-5*. It is clear that the genetic markers for *Ty-2*, *Ty-4*, and *ty-5* resistance developed in this study are effective in identifying their corresponding resistance genes.

### 3.2. Comparison of Outcomes of Natural and Artificial Infection

The ultimate goal of TYLCV resistance breeding, as in any other resistance breeding program, is to accomplish durable and reliable resistance in open field conditions. TYLCV infection is orchestrated by triangular plant-whitefly-TYLCV interactions in nature. Because TYLCV is strictly a whitefly-transmitted virus, there are technical restrictions with respect to manipulating TYLCV infections and maintaining TYLCV-infected tomatoes. TYLCV viruliferous whitefly-mediated inoculation has been widely deployed and facilitates simultaneous monitoring of disease-limiting factors, TYLCV-plant interactions, and insect-plant interactions [34,35,36,37,38,39,40] However, numerous biotic or abiotic factors can interfere with the inoculation process. In this study, natural infection in the field, and artificial inoculation with infectious TYLCV clones in a growth chamber, were optimized and compared. It is impossible to control the amount of virus transmitted by whiteflies or the number of whiteflies feeding on each plant. However, we believe that the outcome of the natural infection experiments is meaningful in evaluating resistance levels because it reflects natural TYLCV transmission and infection processes. Artificial inoculation using infectious TYLCV clones in a controlled environment was conducted separately, and the outcomes from the two different approaches to infection were compared. Symptom development was observed in the entire bodies of the susceptible plants under field conditions but was relatively restricted in newly emerged tissue after agro-mediated inoculation of the apical tissue. In general, susceptible plants in the field developed severe symptoms in the whole plant body when compared with plants of the same genotype that were artificially inoculated and maintained in a growth chamber. The differences in symptom development were probably due to differences in environmental conditions and inoculation processes, so a different disease evaluation scale was used for each condition.

Artificial inoculation with infectious TYLCV clones mediated by *Agrobacterium* is widely used [41,42,43]. However, this approach has limitations because it omits numerous factors affecting viral transmission in nature. All of the genotypes expected to be susceptible to the virus showed disease symptoms under both means of infection, and most genotypes categorized as resistant in the artificial inoculation condition were resistant in natural infection conditions in the field. Although relatively severe disease symptoms in the susceptible genotypes were visually detectable in the field, the window for resistance levels was more traceable under artificial conditions in this study. For instance, the development of detectible symptoms occurred in several resistant commercial cultivars heterozygous for the *Ty-1* resistance gene, although it was not statistically significant in most cases. In the case of TY172 carrying *ty-5* resistance, no symptoms were observed in natural infection, despite significant amount of viral DNA being detected. A small amount of viral DNA and symptom development was detected for TY172 following *Agrobacterium*-mediated inoculation. It appears that *ty-5* resistance alone may not be a stable or reliable source for TYLCV resistance under field conditions compared to the other resistance genes with dominant inheritance. However, further investigation is required to explore the molecular mechanisms involved in viral multiplication without symptom development in the TY172 genotype. In this study, we did not observe *Ty-1* or *Ty-2* resistance under field conditions in Daegu, Korea from June to August 2018.The study of a larger number of genotypes with various combinations of TYLCV resistance, exposed to various environmental pressures, will produce a better understanding of each mechanism of resistance, and resistance conferred by gene-pyramiding under field conditions.

### 3.3. Investigation of the Phenotypes of TYLCV Resistance and Their Combinations

We performed quantitative infectivity assays using both natural infection in the field and artificial inoculation with infectious TYLCV clones in a growth chamber for cultivars with thirty-two diverse genotypes. Ten out of 17 accessions and 8 out of 15 commercial varieties carried at least one gene for resistance to TYLCV. Based on both symptom development and viral accumulation, resistant genotypes in breeding lines tended to display more resistance than the resistant commercial cultivars, although the difference was not statistically significant. Most of the resistant breeding lines used in this study were homozygous for both *Ty-1* and *Ty-2*, although KNU-17 and KNU-19 were only homozygous for *Ty-2*. Most of the resistant commercial cultivars were heterozygous for *Ty-1*, except ‘Oyama’, which was heterozygous for *Ty-2*. Considering the diverse genetic backgrounds of the genotypes used in this study, these results suggest that either the number of resistance genes or the homozygosity for the resistance alleles determines the levels of TYLCV resistance. Although both the *Ty-1* and *Ty-2* loci clearly show dominantly inherited resistance [17,31], it is possible that subtle differences may exist between homozygous and heterozygous resistance. Such a possibility has been suggested for potyviral resistance in peppers [44]. It is also possible that other host factors, in addition to the known major TYLCV resistance genes in these genotypes, affect viral resistance. An improvement of resistance levels resulting from gene-pyramiding has been postulated, and the strongest resistance in both field and artificial infections was observed in breeding lines carrying both *Ty-1* and *Ty-2* resistance. The identification of TYLCV isolates overcoming *Ty-1* or *Ty-2* resistance, or environmental conditions facilitating a TYLCV outbreak, are needed to confirm the effectiveness of gene-pyramiding for TYLCV resistance. The exploration of novel sources of resistance, and analysis of the effects of combining multiple TYLCV resistances, are necessary in order to achieve durable and stable resistance. We are in the process of pyramiding TYLCV resistance for maximum protection against TYLCV.

We performed quantitative infectivity assays using both natural infection in the field and artificial inoculation with infectious TYLCV clones in a growth chamber for cultivars with thirty-two diverse genotypes. Ten out of 17 accessions and 8 out of 15 commercial varieties carried at least one gene for resistance to TYLCV. Based on both symptom development and viral accumulation, resistant genotypes in breeding lines tended to display more resistance than the resistant commercial cultivars, although the difference was not statistically significant. Most of the resistant breeding lines used in this study were homozygous for both *Ty-1* and *Ty-2*, although KNU-17 and KNU-19 were only homozygous for *Ty-2*. Most of the resistant commercial cultivars were heterozygous for *Ty-1*, except ‘Oyama’, which was heterozygous for *Ty-2*. Considering the diverse genetic backgrounds of the genotypes used in this study, these results suggest that either the number of resistance genes or the homozygosity for the resistance alleles determines the levels of TYLCV resistance. Although both the *Ty-1* and *Ty-2* loci clearly show dominantly inherited resistance [17,31], it is possible that subtle differences may exist between homozygous and heterozygous resistance. Such a possibility has been suggested for potyviral resistance in peppers [44]. It is also possible that other host factors, in addition to the known major TYLCV resistance genes in these genotypes, affect viral resistance. An improvement of resistance levels resulting from gene-pyramiding has been postulated, and the strongest resistance in both field and artificial infections was observed in breeding lines carrying both *Ty-1* and *Ty-2* resistance. The identification of TYLCV isolates overcoming *Ty-1* or *Ty-2* resistance, or environmental conditions facilitating a TYLCV outbreak, are needed to confirm the effectiveness of gene-pyramiding for TYLCV resistance. The exploration of novel sources of resistance, and analysis of the effects of combining multiple TYLCV resistances, are necessary in order to achieve durable and stable resistance. We are in the process of pyramiding TYLCV resistance for maximum protection against TYLCV.

The commercial cultivars ‘TitiChal’, which carries *Ty-1*, and ‘Oyama’, which has *Ty-2* resistance, showed no disease symptom in either natural infection (Figure 6C) in the field or artificial infection in the growth chamber, while most of the other commercial cultivars showed low levels of disease symptoms in both conditions. This result implies that, even though *Ty-2* resistance is not as widely deployed in commercial breeding programs as *Ty-1*, *Ty-2*, resistance could also be an effective source of resistance for TYLCV control, at least in Korea, with very limited variation in TYLCV isolates. The effectiveness of *Ty-2* resistance in commercial breeding programs should be further investigated using various TYLCV isolates under different environmental conditions. Although *Ty-4* has been reported to provide resistance additional to the resistance conferred by *Ty-3* [21], an increased level of resistance conferred by *Ty-4* was not detectable in this study. LA4440, which carries *Ty-4* in addition to *Ty-1/3*, developed low levels of disease symptoms, and this phenotype was indistinguishable from that conferred by *Ty-1* alone.

Variations in the symptom development and viral accumulation were observed in plants carrying no known resistance genes. Even though it is difficult to say whether the levels of symptom development are strictly correlated with the levels of viral accumulation, some correlation between symptom development and viral accumulation was apparent. For example, the genotypes with the highest levels of viral accumulation, E6203 and ‘Mini Heuk Su’, showed more severe symptoms than the other susceptible genotypes, although this difference was not statistically significant. In the susceptible genotypes LA1589 (*S. pimpinellifolium*) and Hawaii7998, relatively low levels of symptoms were observed, while viral accumulation was detectable, supporting the hypothesis that other genetic factors limiting viral symptom development exist in these genotypes. It has been reported that there is an accession in *S. pimpinellifolium* that is effective in reducing viral spread, presumably by restricting the transmission of TYLCV via whiteflies [39]. Hawaii7998 is an accession widely known for conferring bacterial resistance, especially to *Xanthomonas* [45], although its connection with the restriction of the development of viral symptoms has not yet been explained. Further analysis should be performed, using plants with a larger number of genotypes grown under diverse environmental conditions, to provide a fuller understanding of the factors affecting viral resistance in tomato crops.

## 4. Materials and Methods

### 4.1. Plant Materials and Marker Analysis

For disease evaluation of *Agrobacterium*-mediated inoculation, 18 accessions and 14 commercially available cultivars were used (Table 1). Genomic DNA was extracted from young leaves, using the CTAB method for marker analysis [46].

Polymerase chain reaction (PCR) (SolGent Co., Ltd., Daejeon, Korea, Cat No. SET15-R500) was performed using 1 µL DNA with 300 ng, 2.5 µL 10X *e-Taq* reaction buffer, 0.5 µL 10 nM dNTP mix, 0.125 µL *e-Taq* DNA polymerase, 18.875 µL ddH_2_O, and 1 µL of 10 pmol of each of the forward and reverse primers. The reactions were carried out in a T-100^TM^ Thermal Cycler (BIO-RAD, Hercules, CA, USA) under the following conditions: initial denaturation at 95 °C for 3 min; 35 cycles of 30 s at 95 °C, 30 s at 55 °C (*Ty-1/3*, *Ty-2*, *Ty-4*, *ty-5*), and 1 min at 72 °C; and a final extension step at 72 °C for 5 min. The restriction enzyme reaction was conducted using 5 µL PCR product, 0.1 µL enzyme buffer, 3.9 µL ddH_2_O, and 1 µL restriction enzyme (10 unit/µL) for 4 h at 37 °C, and the digested fragments were resolved on 2% agarose gels. Detailed conditions for marker analysis are given in Table 2.

### 4.2. Virus Inoculation

Viral infection was performed using two processes, natural and artificial infection, using infectious clones. For natural infection by whitefly, 18 cultivars with three different plants of each genotype were used. Five-week-old plants grown in a greenhouse were transplanted to a field in Kyungpook National University (Daegu, Korea) and grown from mid-June to August 2018, a period of 42 days. Plants were distributed according to a randomized design developed using R studio (R studio, Boston, MA, USA). Each row represented a replicate of the experiment, and three plants of each genotype were distributed at random in the field. To confirm the natural infection, the susceptible control plant genotype M82 was used.

For artificial infection, *Agrobacterium*-mediated inoculation of infectious clones was performed. *Agrobacterium tumefaciens* (GV3101) containing pCAMBIA3301-TYLCV was kindly provided by Prof. Suk Chan Lee, Sungkyunkwan University (Suwon, Korea). TYLCV-IS isolate was used for generating infectious clones. *Agrobacteria* were grown on Luria-Bertani (LB) solid selection medium for 48 h at 28 °C in the dark, and sub-cultures were grown in LB liquid selection medium in the dark for 48 h in a shaking incubator at 28 °C. Rifampicin (100 μg mL^−1^) and kanamycin (50 μg mL^−1^) were used for antibiotic selection. Cultured agrobacterium was centrifuged, and the pellets were diluted to OD_600_ = 0.5 with the suspension buffer [47]. Plants were grown in a growth chamber at 28 °C with a relative humidity of 70% and 16 h of light. Five-week-old tomato plants were used for inoculation. At least three independent inoculations were performed for each plant genotype. Injection and pinprick inoculations were simultaneously performed at the same site on each inoculation plant. Using an ultra-fine 0.5 mL syringe (Becton Dickinson Company, Franklin Lakes, NJ, USA), 50 µL of inoculum was injected into the apical site of a second branching point of a stem, and another 50 µL of inoculum was placed on the branching point. Before placing the inoculum, the site was perforated 3–5 times with a syringe.

### 4.3. Analysis of Disease Severity

Two different experiments involving agro-mediated inoculation and field infection were used for two different scales of disease evaluation. Disease severity in plants grown in the field was evaluated six weeks after natural infection using a disease severity index (DSI) ranging between 0 and 3 [48]: 0 = no visible symptom; 1 = light leaf yellowing of the leaflet margins; 2 = moderate plant stunting with leaf yellowing and curling; and 3 = severe plant stunting with leaf curling and yellowing and cessation of plant growth (Figure 6A).

Disease severity of artificially infected plants maintained in the growth chamber was evaluated five weeks after *Agrobacterium*-mediated inoculation using a previously described 0–4 DSI scale [30]; 0 = no visible symptom; 1 = very slight yellowing of leaflet margins of apical leaves; 2 = some yellowing and minor curling of leaflet ends; 3 = a wide range of leaf yellowing, curling, and cupping; 4 = severe symptoms and stunting. No stunting was observed five weeks after inoculation under growth chamber conditions. The score of inoculation presented represents the average of each cultivar (Figure 7A).

### 4.4. Virus Accumulation Test

To quantify virus accumulation, quantitative real-time PCR (qPCR) was performed. For qPCR, the forward primer TYLCV-IS 1678F and the reverse primer TYLCV-CONS 1756R were used (Supplementary Table S1), as described by Powell et al. [49]. DNA was diluted to 50 ng/µL. The actin gene was used as a reference. PCR was carried out in 10 µL reactions with Power SYBR Green PCR master mix (Thermo Fisher Scientific, Waltham, MA, USA) using a Bio-Rad CFX connect (BIO-RAD, Hercules, CA, USA) according to the manufacturer’s three-step protocol. The amounts of viral DNA were calculated using the delta-delta Ct method.

### 4.5. Statistical Analysis

Duncan’s multiple range analysis was carried out to analyze the TYLCV resistance of the different genotypes used in agro-mediated inoculation and quantitative PCR for virus accumulation tests, using IBM SPSS 25 (IBM, Armonk, NY, USA). The average disease evaluation scores of each trial were used for statistical analysis, and cultivars that lacked the appropriate number of replications were excluded from the analyses.

## Figures and Tables

**Table 1:** Plants used in disease severity analysis.

No.	Plant Information			
	Name	Type	Species	Source ^*b*^
1	M82 *^a^*	Accession	*S. lycopersicum*	TGRC
2	Ailsa Craig		*S. lycopersicum*	
3	E6203 *^a^*		*S. lycopersicum*	
4	Hawaii7998 *^a^*		*S. lycopersicum*	
5	Heinz 1706		*S. lycopersicum*	
6	LA1589		*S. pimpinellifolium*	
7	Black Cherry		*S. lycopersicum*	
8	Money Maker		*S. lycopersicum*	
9	LA4440		*S. chilense*	
10	TY172 *^a^*	Accession	*S. peruvianum*	Volcani center
11	KNU-2 *^a^*		*S. lycopersicum*	AVRDC
12	KNU-4 *^a^*		*S. lycopersicum*	
13	KNU-5 *^a^*		*S. lycopersicum*	
14	KNU-7 *^a^*		*S. lycopersicum*	
15	KNU-9 *^a^*		*S. lycopersicum*	
16	KNU-10 *^a^*		*S. lycopersicum*	
17	KNU-17 *^a^*		*S. lycopersicum*	
18	KNU-19 *^a^*		*S. lycopersicum*	
19	Miniheuksu	Commercial cultivar	*S. lycopersicum*	Asia seed
20	Benekia220 *^a^*		*S. lycopersicum*	Bunong seed
21	TYEndolphin		*S. lycopersicum*	Bunong seed
22	Shinchunggang		*S. lycopersicum*	Farm Hannong
23	Super Dotaerang		*S. lycopersicum*	Koregon
24	TitiChal *^a^*		*S. lycopersicum*	Nongwoo bio
25	TYAltorang *^a^*		*S. lycopersicum*	Nongwoo bio
26	TYSenseQ *^a^*		*S. lycopersicum*	Nongwoo bio
27	Dotaerang Red		*S. lycopersicum*	Sakata seed
28	Oyama *^a^*		*S. lycopersicum*	Sakata seed
29	Dafnis		*S. lycopersicum*	Syngenta
30	B Blocking		*S. lycopersicum*	Takii seed
31	Dotaerang Dia		*S. lycopersicum*	Takii seed
32	Dotaerang TY Winner *^a^*		*S. lycopersicum*	Takii seed

*^a^* Plants used in both natural infection and agro-mediated inoculation. *^b^* TGRC: Tomato genetic resource center at UC Davis, AVRDC: Asian Vegetable Research and Development Center. Other annotations are names of companies and countries.

**Table 2:** DNA markers used in this study.

Locus	Primer Name		Sequence (5′-3′)	Annealing Temp (°C)	Product Size (bp)	Type (Enzyme)	Reference
*Ty-1/3*	14IY218	F	ATG AAG ACA AAA ACT GCT TC	55	R : 383, 226	CAPS (*Ssp*I)	Jung et al., 2015
		R	TCA GGG TTT CAC TTC TAT GAA T		S : 609		
*Ty-2*	20IY10	F	GTT CTA TCA CAA GAC TTG CCA	55	R : 738	Indel	In this assay
		R	TGC ATT CAC CAT TGA TGT ATA AGA		S : 600		
*Ty-4*	18IY23	F	AGA AGA AAT CCA AGA AAA GCA ATA AGA ATG AGG CC	55	R : 304	dCAPS (*Stu*I)	In this assay
		R	CTT GTA ATC ACG TCC ACA ACG		S : 269, 35		
	18IY13	F	CTT CTG TTC TAT GCA GGT GTG	55	R : 228	Indel	
		R	GGA TAC AAC TGT CAA CGC AC		S : 200		
*ty-5*	14IY5	F	TTC AAG TCC TTC TTC AAC	55	R : 300	dCAPS (*Rsa*I)	In this assay
		R	ATA GAT TTA AAC AAC AAT TAT AGA GAT AAA AAA GTT ACC TGT		S : 260, 40		

## Supplementary Materials

The following are available online at https://www.mdpi.com/2223-7747/10/1/9/s1: Table S1: Primers used for quantitative real-time PCR, Figure S1: Sequence data of Solyc11g019800 of the susceptible and resistant haplotypes, Figure S2: Application of gene-specific markers developed in this study.
