# Supplementary material for: Development and Application of Gene-Specific Markers for Tomato Yellow Leaf Curl Virus Resistance in Both Field and Artificial Infections
Source: Plants (Basel). 2020 Dec 23;10(1):9. doi: 10.3390/plants10010009 (PMC7824369; doi:10.3390/plants10010009)
Supplement: Supplementary file 1 [file plants-10-00009-s001.pdf]

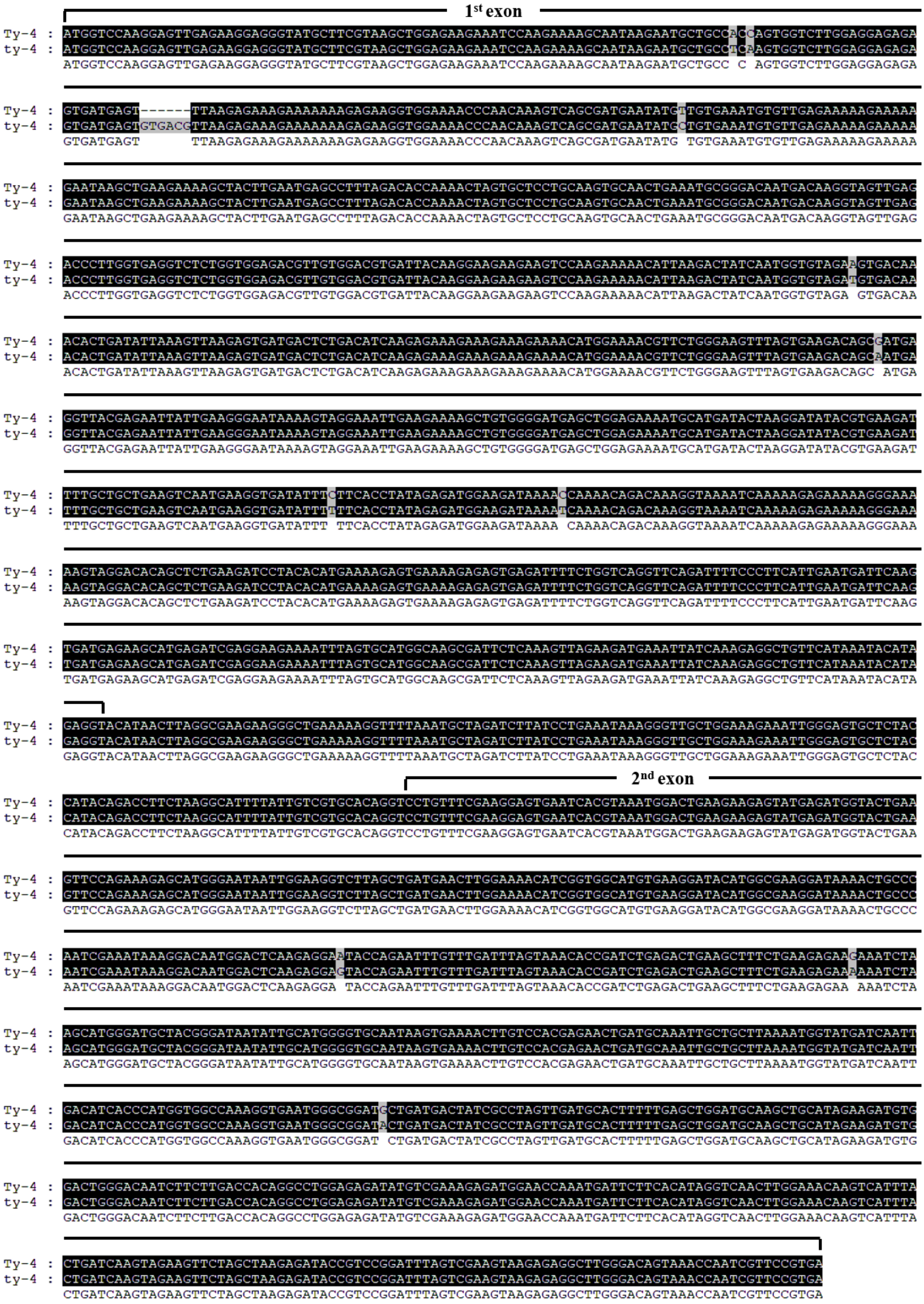

Supplementary Figure 1. Sequence data of Solyc11g019800 of the susceptible and resistant haplotypes.

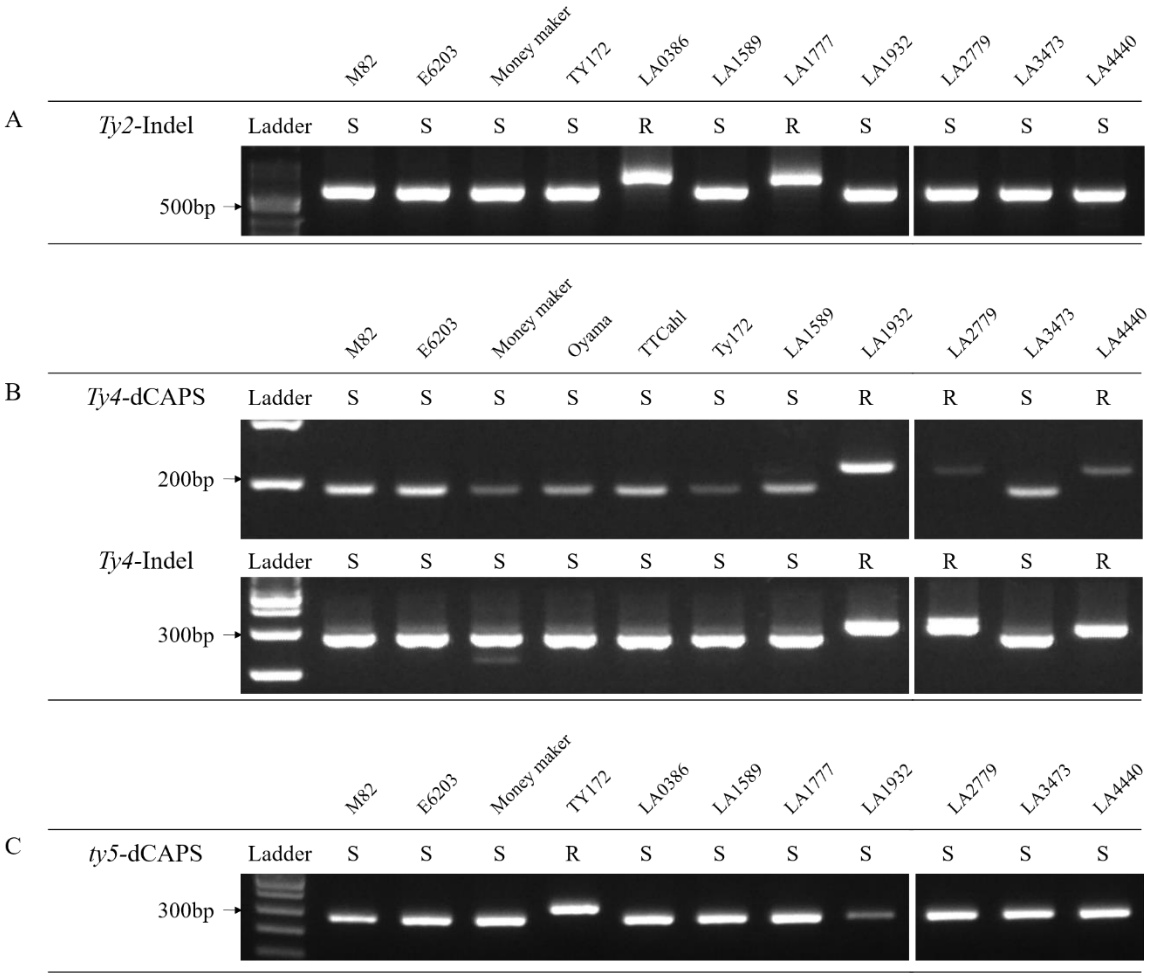

Supplementary Figure 2. Application of gene-specific markers developed in this study. Inbred lines, commercial cultivars and wild species with known TYLCV resistance genes are included. R indicates resistance, and S indicates susceptibility. A: Genotyping result using a gene-specific *Ty-2* marker. B Genotyping results using two *Ty-4* markers. C: Genotyping result using gene-specific *ty-5* marker.

Supplementary Table 1. List of primers used for quantitative real-time PCR.

Primer name	Sequence (5' - 3')	Reference
TYLCV-IS 1678F	CGT CTA GAT ATT CCC TAT ATG AGG AGG T	Powell et al., 2012
TYLCV-CONS 1756R	GGC AAG CCC ATT CAA ATT AAA G	
Actin F	ATA TGC GTC GTT GGC AGA TTG CTG	Anbinder et al., 2009
Actin R	AAC CAA CGG TTC TTC GAC CTG GTA	

11
